# AI based tool for monitoring intensity and fatigue in elite women handball

**DOI:** 10.3389/fspor.2026.1784265

**Published:** 2026-03-04

**Authors:** Florian Felice

**Affiliations:** Department of Mathematics, University of Luxembourg, Esch-sur-Alzette, Luxembourg

**Keywords:** artificial intelligence (AI), explainable AI, handball, machine learning, wearable sensors

## Abstract

We propose an AI-based tool to predict and monitor Key Performance Indicators (KPIs) for player’s activity such as running distance and speed from wearable devices. These KPIs serve as proxies for intensity and fatigue levels in professional athletes. Applied to a women’s professional handball team competing at the EHF Champions League level, our model helps predict player workload and physiological stress, enabling accurate monitoring of player condition. By combining predictive accuracy with explainability methods, our tool not only forecasts fatigue and intensity metrics but also provides actionable insights for coaching staff to optimize training and lineup strategies. This work demonstrates the potential of advanced machine learning methods and can be extended to the prediction of any physiological KPI to support handball performance monitoring.

## 1 Introduction

### 1.1 Motivation

Over the last few years, elite team sports have increasingly embraced data-driven approaches to optimize player performance, reduce injury risk, and enhance tactical decision-making. Today, the sports analytics literature on wearable sensors data is rich (1). Several sports such as basketball, american football, soccer or rugby have fully adopted these technologies (2–5).

As the technology becomes more affordable and the need to include advanced analytics to optimize elite performances increases, handball teams and federations started to include video analytics and wearable sensors as part of the preparation for competitions. The European Handball Federation (EHF) has been at the forefront of this approach by including wearable sensors analysis during the recent european championships competitions (6). However, despite the growing adoption of wearable sensors in team sports, research on predictive models for monitoring performance KPIs from sensor data remains limited, particularly in handball where the literature is scarce beyond descriptive analytics (6). While machine learning has been successfully applied to injury prediction and match outcome forecasting in various sports (1), there is a gap in developing predictive tools that combine forecasting capabilities with explainability to make wearable sensor data actionable for coaches. Our work addresses this gap by proposing an AI-based approach that not only predicts key performance indicators but also provides explainable and actionable insights to support data-driven decision-making in handball.

Elite European teams such as Metz Handball (France) strive to win the world’s most prestigious competition, the EHF Champions League. To reach this goal, the team and staff constantly try to reinvent the way they work and include new tools and technologies in their daily work to push the boundaries of performance. In this context, we collaborate with the team to support them in designing tools from data they extract from wearable sensors. These tools aim to help the team in their quest for additional trophies and titles.

### 1.2 Wearable sensors

Our data are extracted from historical sensor data worn by players from the start of the 2024/2025 season. The gyroscopic sensors are worn on the upper back by players during team trainings and matches. The sensors collect information about movements in 3D space and record directions, speed and intensity of movements and jumps. The collected data are then summarized by session (one match or one team training) and constitute a series as more sessions get added.

Our data set contains a total of 1,617 sessions (108 sessions per player on average) over the 2024/2025 season. One session corresponds to either a match or a team training. The key KPIs of interest for our forecast are the total distance run during the session, the maximum speed, maximal metabolic power and the accumulated acceleration load.

The sensors are worn by the wing, back and pivot players during the sessions. After each session, the staff retrieves the data through the Kinexon API (sensor provider), which automatically formats and summarizes the metrics per session and player for our analysis and modeling.

### 1.3 Paper overview

Working directly with Metz Handball’s staff, we developed a practical machine learning approach for predicting player activity metrics from wearable sensor data. Our framework combines predictive modeling with explainable AI techniques to create actionable insights for coaching decisions. The solution is deployed and leveraged for matches including the local French championship and the EHF Champions League.

In this document, we provide an evaluation of multiple modeling approaches for forecasting a set of performance indicators such as running distance, maximum speed, and metabolic power. Beyond predictive accuracy, we integrate explainability methods to highlight the most influential patterns on future predictions. Such explanations allow coaches to understand both what the model predicts and why.

The rest of the document is organized as follows. Section 2 describes the methodology including data processing, baseline models, the proposed architecture and the explainability techniques used. Section 3 presents the predictive performance and insights from the explainability layer. Section 4 discusses with prior handball-match prediction work and outlines future directions. Finally, Section 5 concludes the paper.

## 2 Methodology

In this section, we describe the methodology used in our study, including data collection, preprocessing, and modeling techniques.

### 2.1 Data analysis & preparation

Our primary data analysis, summarized in Table 1, highlights expected performance patterns across positions. These reflect the physiological demand and specific roles for each players in handball.

**Table 1:** Average values per position for key performance metrics.

Position	Distance	Max speed	Metabolic power	Acceleration load accumulated	Jumps	Changes of direction
	(m)	(km/h)	(W)			
Wing	5294.5	26.1	5560.8	604.9	17.6	55.9
Back	4392.9	24.7	8173.6	540.2	14.2	35.2
Pivot	3964.4	22.4	4388.9	532.1	14.5	22.9
*Team total*	*4356.3*	*24.0*	*4935.3*	*557.7*	*15.2*	*34.6*

Italic values correspond to the average values across the team and all mentioned positions.

We first note that the positions displayed are for Wing players (left and right combined), Back players (left, center and right combined) and Pivot (line player). Goalkeepers are not included in this analysis as no data is available since they do not wear sensors. Indeed, given their specific role, the staff considered (after several experiments) that the data collected from goalkeepers were not relevant and not adequate to monitor any form of performance.

The first pattern is highlighted by the larger total distance run by wing players (5294.5 m) as well as the higher average maximum speed compared to other positions (26.1 km/h). This reflects the specific position on the court which requires longer distances to run as well as their strategic role in fast breaks. The high total distance and maximum speed also illustrate one of the key differentiators for Metz Handball across the European stage with high intensity and multiple fast breaks, primarily driven by their wingers.

Back players show an important difference on the maximum metabolic power measured during a session (8173.6 W). This translates the specific activity of the central players in defense as well as in attack. Indeed, back players are often having lots of contacts with high intensity on both sides of the court. Additionally, the average maximum speed (24.7 km/h) and total distance (4392.9 m) also suggest that back players also play a key role in turnover situations, especially with dynamic center back players such as Petra Vamos and Léna Grandveau.

Finally, we can observe that pivots (line players) show less changes of directions or exertions than wing or back players. This may suggest that the role is more “static.” However, their central role in defense and the positioning in attack suggest less distance to run compared to other players and the positioning may be, from a sensor perspective, considered as less intense than other roles.

Some of these indicators are also recorded and analyzed on the international stage. The analysis of the 2024 European Championships highlighted the swiss player Mia Emmenegger recorded an average maximum speed of 28.28 km/h, the highest from the competition. As a comparison, the average maximum speed across the whole season for Metz is 28.03 km/h for Chloé Valentini, in similar high standards. Additionally, the maximum top speed in that season was recorded at 30.24 km/h, also by wing player Chloé Valentini.

### 2.2 Baseline models

A first approach to model player’s workload is to leverage classical time series techniques and predict each KPI individually. We establish performance benchmarks using three classical time series forecasting methods: an simple moving average, an autoregressive moving average (ARIMA) model, and an exponential smoothing model. The Moving Average predicts the next time stamps as the mean of the last k observations, providing a simple trend-following baseline. The ARIMA (AutoRegressive Integrated Moving Average) model extends the moving average with an autoregressive step to capture linear dependencies and seasonal patterns. Finally, Exponential Smoothing applies exponentially decreasing weights to historical observations, putting more emphasis on recent data.

These univariate approaches serve as reference models for comparing our proposed approach.

### 2.3 Proposed architecture

**Figure 1:**
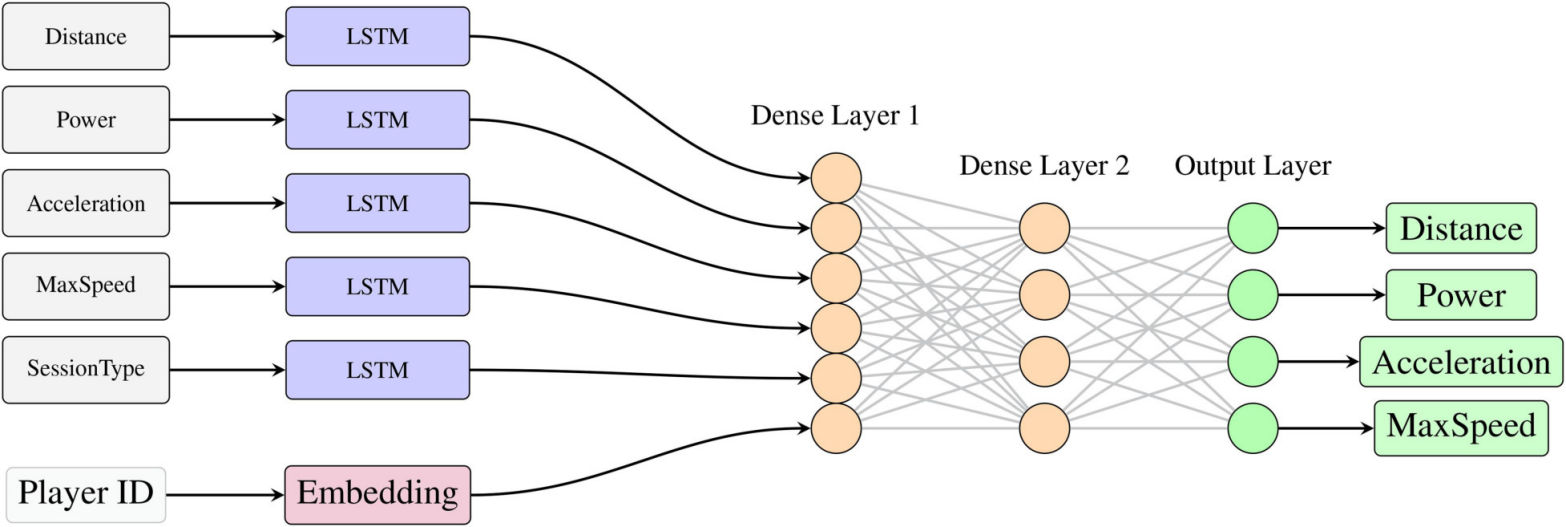
Model architecture with LSTM cells.

Our proposed approach depicted in Figure 1 leverages a recurrent neural network with LSTM cells (7) as a multi-target approach to predict multiple series. Each predicted series corresponds to a specific KPI for an individual player. We thus have as many targets to predict as input series. We also add another series describing the type of session (match or training) as an exogenous input. This series will not be predicted by the model and will help capture specific patterns. Finally, we include an embedding layer to learn the individual representation of the players (position, individual abilities, etc.). The input series and the embedding layer are then concatenated and feed a fully connected network.

We note that our approach allows to include static information (such as player information) with a dense input layer that can be merged with the other inputs in a similar manner. Additionally, the hyper-parameters of our network (such as number of LSTM cells, width and depth of the fully connected network, etc) are optimized using some hyper-parameter optimization (8).

Our approach also includes drop out for the LSTM cells and dense layers. This approach is used to add regularization to the model in the training step but is also used to measure the uncertainty during the predictions. Indeed, we leverage the Monte-Carlo Dropout (MC Dropout) methodology (9) to measure the model’s uncertainty and derive confidence intervals.

### 2.4 Explainability layer

To provide the staff with actionable insights from model predictions, we implement gradient-based feature importance analysis. This technique computes the sensitivity of predictions to input features by calculating gradients with respect to each biomechanical variable. The resulting importance scores reveal which player metrics most influence the performance forecasts for each KPI.

We leverage a large language model (LLM) using the Ph4-mini reasoning model (10) to transform these technical importance scores into natural language explanations. The LLM generates contextual insights that the staff can directly interpret and act upon. For instance, when the model predicts decreased power output, the system might explain that elevated fatigue indicators in recent time steps are the primary contributing factors, suggesting specific intervention strategies.

## 3 Results

### 3.1 Predictive performance

We evaluate our proposed LSTM-based approach against the presented baseline models to assess predictive accuracy across our target KPIs. We predict the next 10 sessions for each player on our test dataset. We present the Root Mean Square Error (RMSE) results across each KPI in Table 2. Our LSTM model consistently outperforms all baseline models across every measured KPI. The greatest accuracy gain is observed for the Distance Total metric, where the LSTM achieves an RMSE of 1273.45 which is 14.0% lower than the best baseline performance.

**Table 2:** Baseline models’ RMSE.

Model/metric	Distance total	Metabolic power max	Acceleration load accumulated	Speed max
Moving average	1480.33	1116.03	164.74	4.28
ARIMA	1515.09	1267.72	168.56	4.03
Exponential smoothing	1514.36	1273.93	175.95	4.03
LSTM (proposed)	**1273.45**	**995.67**	**145.32**	**3.52**

Bold values correspond to best model with lowest RMSE.

We note that the greatest gain is observed for Distance Total which can be explained by the link with the type of session. Indeed, the type of session (match or training) has a direct impact on the total distance run by players. Therefore, our LSTM model is able to capture these patterns by taking these exogenous inputs into account and provide more accurate predictions. Additionally, the multi-target approach allows the model to leverage non-explicit correlations between the different KPIs to improve accuracy.

**Figure 2:**
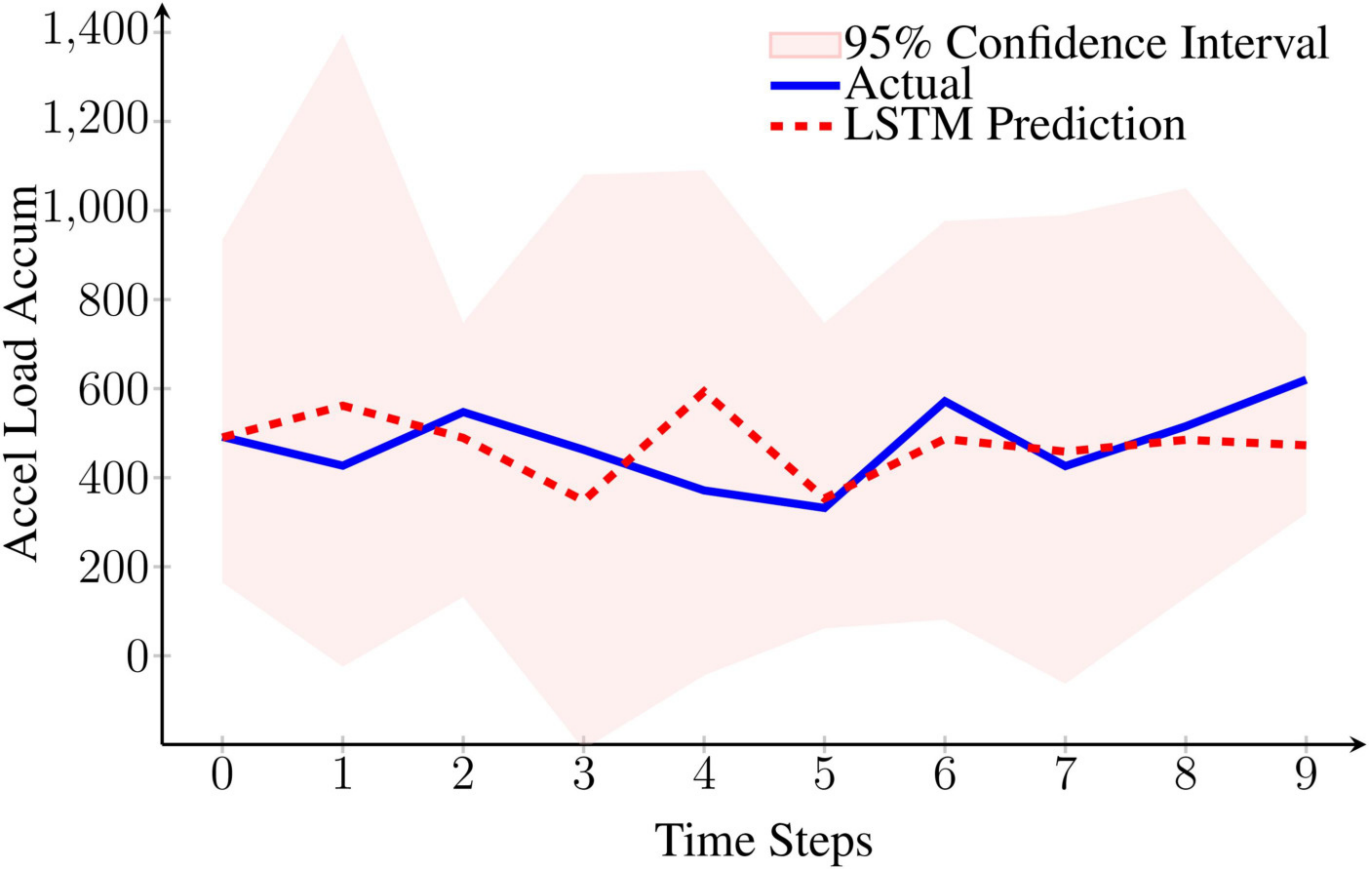
LSTM prediction accuracy for acceleration load accumulation over 10 time steps on the test set. The model achieves RMSE = 107.30 and MAPE = 18.5%. We observe that the actual observations are all within the 95% confidence intervals.

We illustrate in Figure 2 the model’s prediction quality for a randomly selected player, specifically showing acceleration load accumulation forecasts over a 10-step prediction horizon. We can observe that all actual observations fall within the 95% confidence intervals generated through MC Dropout. The confidence level is set by default to 95% and will be adjusted based on empirical feedback from the coaching staff throughout the deployment of the solution.

These confidence intervals will provide valuable information for coaches, as they understand the uncertainty associated with each prediction.

### 3.2 Explainability insights & real-world deployment

The gradient-based feature importance analysis identifies recent training intensity and player position as key drivers for distance predictions, while metabolic power forecasts rely heavily on historical outputs and recovery periods. The Large Language Model (Ph4-mini with reasoning) translates these technical scores into actionable coaching insights, generating natural language explanations highlighting potential player fatigue risks.

The MC Dropout uncertainty quantification allows us to adjust confidence levels in predictions, while the explainability layer provides the coach with insights, essential for adoption in professional sports environments.

## 4 Discussion

### 4.1 Comparison with prior handball-match work

Our work follows a natural continuation from (10). Outside of the technical change from tabular to sequential data, the focus of this work oriented towards workload and physical activity monitoring. The direction of the work still starts backwards from athletes and coaches needs of understanding player’s activity to monitor workload and optimize the team’s performance. We include explainable AI methods as a critical layer to extract model’s learnt patterns and feed into a large language model to translate technical conclusions into sports insights and actions.

While the intent from match prediction and workload monitoring is different, both works have, in their roots, still the same objective: anticipate trends and optimize the team’s performance. Therefore, considering the implications from Section 3.2, the results from this work and match predictions can be used in conjunction and seen as complementary. Match predictions, along with their AI-generated explanations, can be considered as the anticipation of match patterns based on matches history, workload predictions can be used to understand physical constraints. While the predictions explanations will provide trends where some players can be expected to have an important impact on the match, the coach can plan for adapted strategies given the predicted ability for players to put physical intensity.

### 4.2 Future work and potential extensions

This work is done leveraging a specific wearable sensors technology for movement patterns. The developped framework is not restricted to any type of data and can be extended to other sources. In particular, discussions with the handball professionals also suggested the integration of cardiological data to monitor heartbeat and physical intensity. Therefore, our work can be replicated for other sports, sensors technology and even for recreational purposes.

Professional athletes and their staff also monitor health via survey tools. This allows coaches to collect feedback on players’ potential fatigue, injuries or health problems. Furthermore, the menstrual cycle of women’s athletes is also monitored to adapt the physical expectations of players. The addition of such data could constitute a complete set to provide insightful information to the model, and thus to the coach, on how to prepare matches and adapt players workloads.

## 5 Conclusion

We presented an AI-based tool for monitoring player workload metrics from wearable sensor data. The prediction performance of the LSTM step achieved superior performance over classical time series methods across all evaluated KPIs. The integration of explainable AI through gradient-based feature importance and natural language generation enables coaches to understand and act upon model predictions without technical expertise.

The successful deployment at Metz Handball during the 2025/2026 season will demonstrate the practical value of combining predictive accuracy with interpretability in professional sports environments. Beyond its application to handball, our work constitutes a generalizable framework for predictive modeling with explainability that extends to any domain involving time series forecasting for individuals where interpretable insights are essential for decision-making. While we demonstrate the approach with wearable sensors in professional handball, providing coaches with actionable information to monitor player performance, the methodology can be readily adapted to other sports, health monitoring applications, or any field requiring personalized time series predictions with transparent explanations. The tool’s modular design allows extension to other sports, sensor technologies, and physiological data sources, supporting broader applications in sports, ranging from young and recreational athletes to elite performance monitoring injury and optimizing for performance.

## Data Availability

Confidential data shared by the professional team, not available publicly. Requests to access the datasets should be directed to florian.felice@uni.lu.
